# Prevalence and sonographic changes compatible with fatty liver disease in patients referred for abdominal ultrasound examination in Aracaju, SE*

**DOI:** 10.1590/0100-3984.2014.0124

**Published:** 2016

**Authors:** Josilda Ferreira Cruz, Mário Augusto Ferreira Cruz, José Machado Neto, Demetrius Silva de Santana, Cristiane Costa da Cunha Oliveira, Sônia Oliveira Lima

**Affiliations:** 1 Master, Professor, Department of Medicine, Universidade Tiradentes, Aracaju, SE, Brazil.; 2 Graduate Student of Medicine, Universidade Tiradentes, Aracaju, SE, Brazil.; 3 Physician, Fundação Hospitalar de Saúde, Aracaju, SE, Brazil.; 4 Physician, Department of Post-Graduation in Physiological Sciences, Universidade Federal de Sergipe (UFS), São Cristóvão, SE, Brazil.; 5 PhD, Professor, Department of Post-Graduation in Health and Environment, Universidade Tiradentes, Aracaju, SE, Brazil.; 6 PhD, Professor, Department of Medicine, Universidade Tiradentes, Aracaju, SE, Brazil.

**Keywords:** Ultrasonography, Fatty liver, Prevalence

## Abstract

**Objective:**

To estimate the prevalence and evaluate sonographic findings compatible with changes consistent with hepatic steatosis in patients referred for abdominal ultrasonography at four reference centers in Aracaju, SE, Brazil.

**Materials and Methods:**

Prospective, descriptive survey, with analytical and quantitative approach, comprising abdominal ultrasonography scans performed with a convex, dynamic 3.75 MHz transducer. Liver dimensions and parenchymal echotexture were evaluated, classifying hepatic steatosis into grades (1, 2 or 3). The SPSS^®^ 22.0 software was used for statistical analysis, adopting *p* < 0.05 as significance level.

**Results:**

A total of 800 individuals (561 women and 239 men) were evaluated. The prevalence of steatosis was 29.1%, and the male patients were most affected, presenting with more advanced grades of disease (*p* = 0.021), as follows: 119 grade 1 (51.0%); 94 grade 2 (40.4%); and 20 grade 3 (8.6%). The median age patients' was 46 years.

**Conclusion:**

In the present study sample, the prevalence of hepatic steatosis was high, particularly in the male patients. Ultrasonography is suggested as a first choice for the diagnosis of this condition, considering its wide availability, low cost and absence of side effects or risks to the patient.

## Abstract (pt)

**Objetivo:**

Estimar a prevalência e avaliar as alterações ecográficas compatíveis com esteatose hepática em pacientes encaminhados para exame de ultrassonografia abdominal em quatro centros de referência de Aracaju, SE, Brasil.

**Materiais e Métodos:**

Estudo prospectivo, descritivo tipo *survey,* com abordagem analítica e quantitativa, realizado mediante exame de ultrassonografia abdominal com transdutor convexo, dinâmico e com 3,75 MHz. Foram avaliadas as dimensões do fígado e a ecotextura do parênquima, classificando a esteatose hepática em graus (1, 2 ou 3). Considerou-se nível de significância *p* < 0,05 e foi utilizado o programa estatístico SPSS^®^ 22.0.

**Resultados:**

Foram avaliados 800 indivíduos, sendo 561 mulheres e 239 homens. A prevalência de esteatose foi 29,1%, sendo maior em homens. O gênero masculino apresentou graus mais avançados de esteatose hepática (*p* = 0,021). Dos diagnosticados, 119 tinham grau 1 (51,0%), 94 apresentavam grau 2 (40,4%) e 20 tinham grau 3 (8,6%). A mediana de idade foi 46 anos.

**Conclusão:**

A prevalência da esteatose hepática foi alta na amostra analisada, sendo maior no gênero masculino. Sugere-se a ultrassonografia como primeira opção para o diagnóstico desta afecção, por ser um método acessível, de baixo custo e sem efeitos colaterais ou riscos para o paciente.

## INTRODUCTION

Liver steatosis is characterized by deposition of lipids on hepatocytes, exceeding 5% of the total liver weight, in the absence of other causes of hepatic diseases such as viral hepatitis, alcohol consumption and metabolic diseases^(1)^. Such a condition is the most simple component of non alcoholic fatty liver disease (NAFLD), whose spectrum includes from simple steatosis, cirrhosis, to possible progression to hepatocellular carcinoma^(1,2)^. The steatosis prevalence has been increasing worldwide, probably because of changes in lifestyle, eating habits and developments in diagnostic methods^(3)^. The relevance of such a fact increases as one considers that liver steatosis precedes, and many times signal the development of cardiovascular disease, hypertension and type 2 diabetes mellitus, associated with increased mortality^(4,5)^.

Most individuals with NAFLD characterized by simple steatosis are asymptomatic, thus the disease insidiously develops with few reports of bad feeling and abdominal discomfort. Physical examination may be normal, and at most hepatomegaly is observed^(6)^. Routine abdominal ultrasonography (US) reveals much higher prevalence values than those observed in investigations by means of laboratory aminotransferases tests^(7)^. Therefore, US plays a relevant role as a complementary method in the evaluation of liver conditions such as NAFLD, since it allows for an early diagnosis in asymptomatic patients^(8-10)^. With the use of US, the prevalence of steatosis in industrialized countries ranges from 20% to 40%^(11)^.

Computed tomography (CT) has low specificity for the diagnosis of liver steatosis, with high rate of false-positive results, besides its high-cost, poor practicality, and exposure of the patient to ionizing radiation^(7)^. Magnetic resonance imaging (MRI) is considered to be the most effective noninvasive method for the diagnosis of liver steatosis, but is expensive and still not widely accessible in Brazil^(12)^. Contrast-enhanced CT and MRI are superior to US to evaluate the radiological patterns of liver tumors^(13-15)^.

US demonstrates to be a sensitive and relevant method for the diagnosis of abnormalities of the liver, gallbladder and intra-and extrahepatic biliary tract^(16,17)^. The US sensitivity for NAFLD ranges from 60% to 94%, and the specificity, from 88% to 95%^(18,19)^, being the first option for the diagnosis of liver steatosis for its simplicity, low cost, absence of ionizing radiation, wide availability and absence of side effects^(10,20)^.

The present study was aimed at estimating the prevalence and evaluating the sonographic findings compatible with liver steatosis in patients referred for routine abdominal US in four reference centers of the city of Aracaju, SE, Brazil.

## MATERIALS AND METHODS

Prospective, descriptive survey with a quantitative, analytical approach. The data were collected in four US clinics in the city of Aracaju, SE, Brazil, in the period from July 2013 to July 2014, after approval by the Committee for Ethics in Research or Universidade Tiradentes (under No. 010513R). The utilized apparatuses are similar in terms of technological and imaging quality levels, namely, a Voluson 730 Pro (GE Healthcare), a Nemio 17 (Toshiba), a Nemio XG SSA 580 A (Toshiba), and an EnVisor C HD (Philips Healthcare).

The sample calculation took into consideration a prevalence value of 27.3%, according to a study developed by Jeong et al.^(21)^, with an error value corresponding to 5% according to the formula developed by Pocock^(22)^, as follows:

$$E^{2}=\alpha^{2}.p.q/n$$

where: *E* = sampling error; *p* = prevalence; *q* = complementary prevalence; *α* = 1,96. A minimum sample calculation of 304 individuals is obtained and, considering a loss of 10%, a total of 335 is found; however, in the present study, 800 patients were assessed.

The present study included both male and female patients aged between 18 and 60 years, whose alcohol consumption corresponded to < 40 g/day. The following exclusion criteria were adopted: individuals who had already undergone US scan, patients with hepatocarcinomas, other malignant tumors, cirrhosis, referred for management of liver steatosis, or those Who were not able to answer the questionnaire (mental deficiency).

Abdominal US scans were performed with a convex, 3.75 MHz, dynamic transducer (with continuous and automatic imaging). All the scans were performed by a single physician with experience in imaging diagnosis of liver steatosis. The patients were appropriately prepared, i.e., six-hour fasting and use of an antiflatulent agent. The following variables were taken into consideration for the US scan: cover dimensions, liver borders characteristics, echotexture of the parenchyma, and classification of the liver steatosis into grades. The measurements of the liver dimensions were done from the longitudinal diameter on the anterior hemiclavicular line, and the borders were evaluated as either regular or irregular. The parenchymal texture was evaluated as either homogeneous or heterogeneous, and the liver steatosis was classified into grades^(19)^, as follows: 0 - normal echogenicity; 1 - mild steatosis, with visualization of fine echoes from the liver parenchyma, normal visualization of the diaphragm and intrahepatic vessels; 2 - moderate steatosis, with diffuse increase in fine echoes, impaired visualization of intrahepatic vessels and diaphragm; 3 - severe steatosis, with significant increase in fine echoes, with impaired or absent visualization of intrahepatic vessels.

The prevalence was calculated by means of the number of individuals diagnosed with liver steatosis divided by the total number of individuals in the sample, with their respective confidence intervals of 95%. The statistical significance was set in 5% (*p* < 0.05). The Statistical Package for the Social Sciences 22.0 was utilized for statistical analysis.

## RESULTS

**Figure 1:**
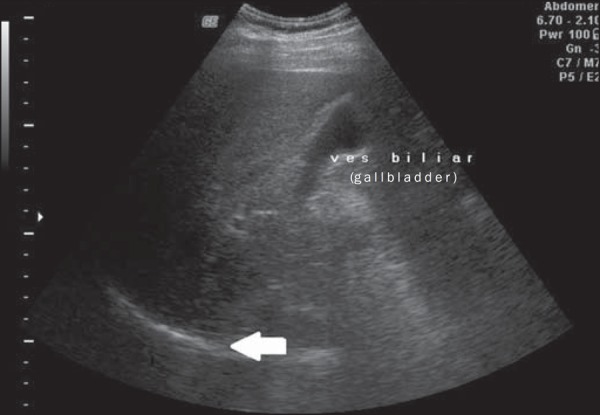
Liver steatosis grade 1. Liver parenchyma with slightly increased echogenicity and normal visualization of the diaphragm (arrow).

**Figure 2:**
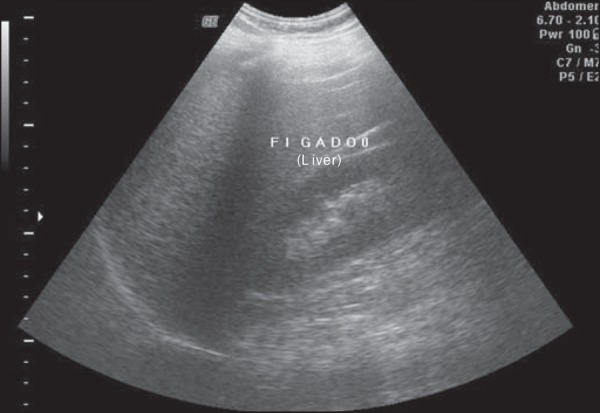
Liver steatosis grade 2. Liver parenchyma with increased echogenicity in relation to the renal cortex. Impaired visualization of intrahepatic vessels.

**Figure 3:**
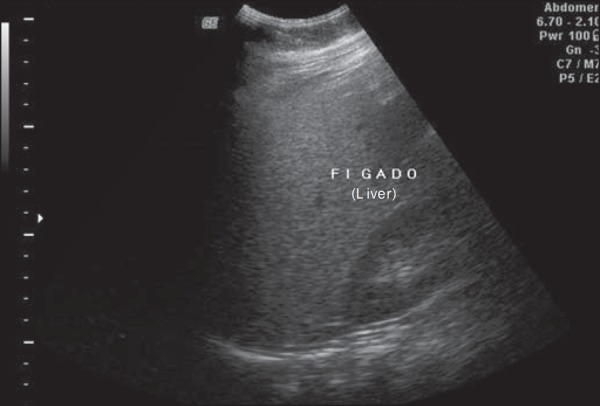
Liver steatosis grade 3. Liver parenchyma with increased echogenicity in relation to the renal cortex. Impaired visualization of intrahepatic vessels and diaphragm, and posterior acoustic shadowing.

A total of 800 individuals (561 women and 239 men) were evaluated. Out of the total sample, 233 (29.1%) patients were diagnosed with liver steatosis, 153 (65.7%) female and 80 (34.3%) male. The prevalence in men was of 33.4%, and in women, 27.2%. As regards grades, 119 patients had grade 1 (51.0%) (Figure 1), 94 presented with grade 2 (40.4%) (Figure 2), and 20 were grade 3 (8.6%) (Figure 3).

Statistically significant association was observed as regards sex and grade of liver steatosis, with men presenting with more advanced grades (*p* = 0.021), and lower than expected number of men for grade 1, and higher than expected number for grades 2 and 3. Women presented a higher than expected number for grade 1, and lower for grades 2 and 3 (Table 1).

**Table 1:** Sex × liver steatosis grades

			Liver steatosis grades		
			Grade 1	Grade 2	Grade 3
			(*n* = 119) 87	(*n* = 94) 57	(*n* = 20) 9
Sex	Female (*n* = 153)	Found			
		Expected	78.1	61.7	13.1
		Percentage	73.1%	60.6%	45.0%
	Male (*n* = 80)	Found	32	37	11
		Expected	40.9	32.3	6.9
		Percentage	26.9%	39.4%	55.0%

**Figure 4:**
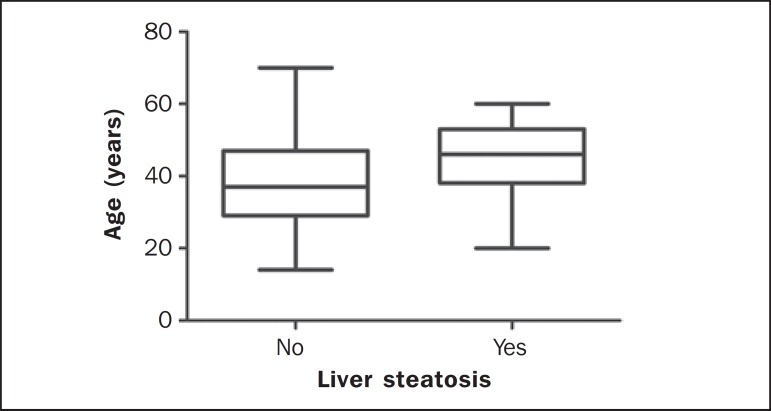
Median age of patients with liver steatosis.

**Figure 5:**
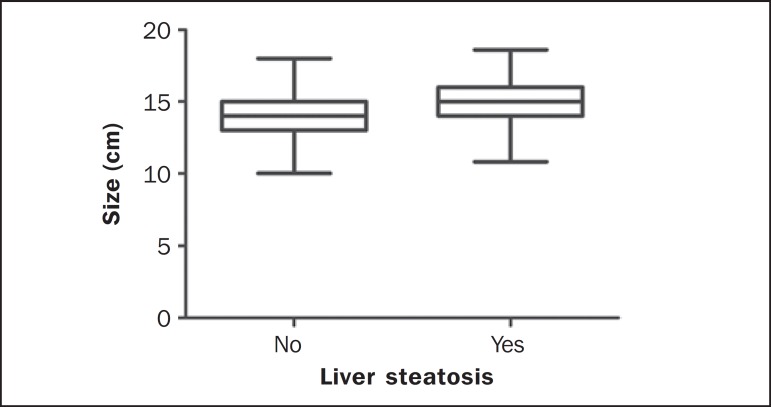
Size of the liver in individuals with and without liver steatosis.

The median age of individuals with liver steatosis was 46 years (1st quartile: 38; 3rd quartile: 53) and 37 years for those who did not have liver steatosis (1st quartile: 29; 3rd quartile: 47), as shown on Figure 4. In the patients with liver steatosis, the median size of the liver was 15.0 cm (1st quartile: 13.8; 3rd quartile: 15.8), and in those with normal sonographic findings, the liver size was 14.0 cm (1st quartile: 13.0; 3rd quartile: 15.0), as shown on Figure 5.

## DISCUSSION

US is a relevant noninvasive method for evaluating the liver, particularly to detect steatosis, since aminotransferases level are not a good parameter to detect NAFLD^(23)^. Imaging methods cannot differentiate liver steatosis from steatohepatitis, so liver biopsy is the only method capable of differentiating among the several NAFLD spectra. Some studies show the relevance of US, proposing that normal or grade 1 hepatic echogenicity rules out the necessity of liver biopsy^(24,25)^. US might become an initial screening tool for the diagnosis of NAFLD^(8,16)^, non transmissible chronic disease, one of the most common diseases nowadays, involving a wide range of factors including the genetic, environmental and metabolic ones^(11)^.

In Brazil, the prevalence of liver steatosis ranges from 18% to 23%^(26,27)^; in Germany, 40%^(28)^; in Italy, 20%^(29)^; in United States, 33%^(30)^; in China, 17.2%^(31)^; in South Korea, 27.3%^(21)^; and in Iran, 21.5%^(1)^. The present study has shown that 29.1% of adult individuals assessed in Aracaju presented liver steatosis. The high frequency of this liver disease in the different countries may justify the inclusion of ultrasonography in the routine complementary investigation aimed at an early diagnosis of liver steatosis.

The images obtained by conventional US did not had an objective or quantitative nature, although the finding of the pattern of a bright liver with increased hepatorenal echogenicity ratio is widely accepted as reliable and sensitive for the presence of liver steatosis^(32)^. US, despite its facility, low cost and absence of adverse effects, is highly dependent on the operator and on the steatosis grade. In the present preliminary, prospective study, the scans were performed by a single and experienced investigator. CT also makes the diagnosis of liver steatosis and also its quantification, but exposes the patient to ionizing radiations, and, similarly to US, its greatest effectiveness is achieved as the steatosis affects more than 33% of the liver^(24)^. FibroScan elastography is another noninvasive imaging method to evaluate liver steatosis, measuring the liver parenchyma elasticity^(33)^. As coupled with MRI, it presents a good diagnostic performance in the detection of advanced fibrosis and cirrhosis, with the advantage of evaluating the elasticity of the whole liver parenchyma, and not only one area of the liver as the FibroScan does^(24)^.

Lankarani et al. have shown that liver steatosis is more prevalent in men than in women, with 26.4% and 17.9%, respectively. Such data are similar to the ones found in the present study, with a male prevalence of 33.4% and female prevalence of 27.2%, although a higher number of women have been evaluated. Probably, this occurs because of the high estrogen levels and low androgen levels present in women before the menopause, thus favoring their hepatic lipid metabolism. This might also be a result from the high androgen levels present in men, favoring the hepatocytes function^(1)^.

Cotrim et al., in a study developed in Brazil, involving 1280 patients, have found a mean age of the individuals with NAFLD of 49.8 years^(27)^. Schild et al. have shown a linear tendency towards increasing the NAFLD prevalence with the increase in the age range^(34)^. In Aracaju, the median age among individuals with non alcoholic liver steatosis was 46 years. Therefore, it is suggested that the use of US for screening for diagnosis, particularly in this age range, allows for actions to minimize the harmful effects of the NAFLD progression.

## CONCLUSIONS

The NAFLD prevalence in the population studied in Aracaju was of 29.1%, being higher in the male population, and the median age among the individuals with steatosis was 46 years. The sonographic findings in the liver were fine echoes from the liver parenchyma, with normal visualization of the diaphragm and intrahepatic vessels; diffuse increase in the fine echoes, with impaired visualization of intrahepatic vessels and diaphragm; and significant increase in fine echoes with impaired or absent visualization of intrahepatic vessels. Such alterations allowed for classifying liver steatosis into grades 1 (mild), 2 (moderate), and 3 (severe), respectively.
